# Mechanical Vibration and Its Therapeutic Application

**Published:** 1905-08

**Authors:** 


**Mechanical Vibration and Its Therapeutic Application.** By **M. L. H. Arnold Snow,** M.D., Professor of Mechanical Vibration Therapy in the New York School of Physical Therapeutics. Octavo, pp. 297. Illustrated. New York: The Scientific Authors' Publishing Company. 1904. (Price, $2.50.)

While of little real scientific value this book finds a place for itself in medical literature because it tells all there is to tell about the latest therapeutic fancy—vibratory treatment. One of its chief claims to distinction is that it is thorough and describes every mechanic vibrator on the market,—and there are plenty of them,—from those which give the patient a light touch, to the more pretentious and serious outfits which bore into the very vitals of the question and make the victim feel like the sphygmographic tracing of a chill. The book explains how vibration acts on various organs. For the amateur vibratory enthusiast it will be of some use, though the anatomic charts showing the nervous system have been reduced in reproduction to such small dimensions as to be wholly incoherent and absolutely worthless. The tabulations of muscles with their origin, insertion and nerve supply are very complete, and show careful encyclopedic study.

